# AUGMENT: Navigation Apps Instruction for Older Adults With Cognitive Impairment

**DOI:** 10.1093/geroni/igab046.735

**Published:** 2021-12-17

**Authors:** Neil Charness, Walter Boot, Sara Czaja, Wendy Rogers, Nicholas Gray, Dorota Kossowska-Kuhn, Michael Pervratil

**Affiliations:** 1 Florida State University, Tallahassee, Florida, United States; 2 Weill Cornell Medicine/Center on Aging and Behavioral Research, New York, New York, United States; 3 University of Illinois Urbana-Champaign, Champaign, Illinois, United States; 4 Psychology Department, Florida State University, Florida, United States; 5 Psychology Department, Tallahassee, Florida, United States

## Abstract

Augmenting User Geocordinates and Mobility by ENhanced Tutorials (AUGMENT) is a development project in the ENHANCE Rehabilitation Engineering Research Center aiming to promote community engagement for aging adults with cognitive impairment (CI) from stroke, traumatic brain injury, and mild cognitive impairment. AUGMENT aims include 1) providing proof of concept that a robust instructional package can support successful use of existing, complex navigation apps, Google maps and rideshare app Uber, by a diverse set of people with CI; and 2) providing proof of product by testing performance with and without instruction. We discuss the needs assessment phase and development of new tests to assess wayfinding abilities and reported difficulties with navigation, using a control sample of 384 community-dwelling older adults. We found that self-reported navigation difficulties are predicted (R-square = .28) by gender, a spatial orientation test, self-reported memory ability, and severity of memory difficulty.

